# Labeling in a Latinx Community: Public Health Implications for Youth and Role in Community Narratives

**DOI:** 10.21203/rs.3.rs-2626181/v1

**Published:** 2023-03-14

**Authors:** Elodia Caballero, Alexandra Minnis, Deepika Parmar, Melissa Zerofsky, Megan Comfort, Marissa Raymond-Flesch

**Affiliations:** University of California, San Francisco; RTI International; University of California, San Francisco; University of California, San Francisco; RTI International; University of California, San Francisco

**Keywords:** Labeling, social connection, adolescent, Latinx, stigma

## Abstract

**Background:**

Youth of color are disproportionately subjected to negative formal and informal labels by parents, peers, and teachers. This study examined the consequences of such labels on health-protective behaviors, wellbeing, peer networks and school engagement.

**Methods:**

In-depth interviews were conducted with 39 adolescents and 20 mothers from a predominantly Latinx and immigrant agricultural community in California. Teams of coders completed iterative rounds of thematic coding to identify and refine key themes.

**Results:**

Dichotomous labeling of “good” and “bad” was pervasive. Youth labeled as “bad” experienced limited educational opportunities, exclusion from peers, and community disengagement. Additionally, preservation of “good kid” labels compromised health protective-behaviors including foregoing contraception. Participants pushed back on negative labeling when it was applied to close family or community acquaintances.

**Discussion:**

Targeted interventions that foster social belonging and connection rather than exclusion may facilitate health protective behaviors and have positive implications for future trajectories among youth.

## Background

Adolescence is a highly dynamic period characterized by rapid physical, cognitive and social developmental changes [1]. Whereas structural determinants of health such as wealth, income, education, health-care access, and employment opportunities, have salient health impacts across an individual’s lifetime, proximal and intermediate determinants, such as social connectedness to peers, families and school are also crucial in shaping health-related behaviors and educational trajectories among youth [2, 3]. Latinx youth and youth in immigrant families face unique challenges in achieving social connectedness under the load of acculturative stress which cumulatively contributes to health disparities [4-6].

Social connections are one of several important factors that protect against poor health outcomes in adolescence. Safe and supportive families, schools, and community as well positive and supportive peers are crucial to helping young people develop to their full potential and attain the best health in adulthood [7]. Likewise, disconnection and exclusion from supportive networks can have detrimental effects [8]. Poverty and socioeconomic deprivation can strain adolescents' opportunities to establish or maintain supportive structures. As demonstrated by Nurd-Sharps and Lewis (2018), youth disconnected from social support and networks are twice as likely to live in poverty [9]. These youth are also at higher risk for undesired pregnancy, incarceration, high school disenrollment and mental health conditions [9-11].

Labeling theory postulates that self-identity can be influenced by terms used to describe or classify people [12]. It argues once individuals are labeled as deviants, their navigation of future scenarios are influenced by these labels [12, 13]. Initial studies of labeling focused on formal labeling imposed by the justice system. However, recent literature underscores the relative importance of informal and perceived labeling [14]. For adolescents, informal negative labeling by the community, family, and peers can be equally or even more impactful on increased recidivism when compared with formal labeling through the justice system [15].

Research, largely focused within the school setting, has shown youth of color are more likely to receive negative social labels for age normative behaviors than white youth, resulting in racial and ethnic disparities in discipline. Morris (2005) showed that Latinx students are more likely to receive some sort of school punishment, oftentimes not consistent with their behavior, in comparison to non-Latinx white students [17]. To this end, school discipline practices can become almost exclusively a form of labeling and social control that disproportionately targets and imposes negative labels on youth of color.

A downfall of labeling is its oversimplicity of the human condition, often enforcing rigid identity binaries and confining narratives. A great deal of literature covers the outcomes of formal and informal labels on youth of color, but very few examine the youth’s perception and internalized understanding of such labels. More so, literature on the subsequent impact on youth’s health and wellness is also scarce. This is critical as the disparate impact and outcomes of exclusionary labels widen existing health disparities for youth of color who already experience higher rates of adverse childhood experiences (ACEs) and other traumas [19].

Understanding how youth perceive and react to harmful labels can help to uncover strategies to mitigate the use of labels in different settings, thereby diminishing their impact on psychological wellbeing and connection to preventive health services.The objective of this analysis is to investigate how community and peer labeling of Latinx adolescents affects their relationships with peers, engagement in school, health behaviors, and support from adults in the primarily Latinx agricultural community of Salinas, CA.

## Methods

### Setting

Salinas, CA is the urban center of the Salinas Valley, the agricultural region on California’s central coast. The agricultural industry is the largest employer in the region. Salinas is 77% Latinx and 38% first generation immigrants [20]. 17% of the population lives below the federal poverty level and the city’s public schools have universal free breakfast and lunch programs due to rates of childhood poverty [21]. Civic engagement and community involvement has been shown to be of high importance among Salinas residents through attendance of public hearings, advisory boards, and community planning meetings to support programs and policies aim to reduce poverty rate ^[21]^.

### Study Population and Design

#### Youth Study Participants:

A Crecer: The Salinas Teen Health Study, is a community-engaged, mixed methods, longitudinal cohort study that followed 599 primarily Latinx adolescents during their transition from eighth grade into high school. Participants were eligible if they were in 8th grade, between 12 and 15 years old, spoke English or Spanish, and planned to live in Salinas for at least one year following enrollment. From November 2015 to March 2017 bilingual and bicultural research assistants enrolled youth from the four middle schools in the Salinas Union High School District. This sample represented approximately one third of all 8th graders in the district. The focus groups and community engagement strategies that informed our recruitment approach and research plans have been published previously [22].

All 599 participants completed interviewer-administered questionnaires with additional data on sensitive topics collected via audio computer-assisted self-interviewing. We invited a subsample of 39 participants (20 girls and 19 boys) to participate in two in-depth interviews (78 interviews total) occurring at approximately months six and eighteen of their study participation. Within each school we randomly selected in-depth interview participants within subgroups defined by self-identified gender, socioeconomic and educational measures. Interview topics included adolescents’ future aspirations, support systems, and relationships. Participants received a $20 payment for each study visit.

#### Mother Study Participants

The Salinas Teen Health research team completed an additional 20 interviews with mothers of youth in the study cohort. Mothers were purposely sampled to include US born and immigrant participants as well as mothers with a variety of educational backgrounds. Sampling was also based on the self-identified gender of the child as well as their immigrant generation status. All interviews with mothers were conducted by bilingual bicultural research assistants in Spanish in accordance with the mothers’ preferences. These interviews focused on mothers’ experiences parenting in Salinas, their goals for the health and wellbeing of their children, and the formal and informal supports that they and their children have within the community.

The RTI and UCSF Institutional Review Boards approved all study activities. Parent permission and minor assent was obtained for all adolescent participants. Mothers provided written informed consent.

### Qualitative Analyses

#### Youth Data

Qualitative analyses were conducted by six team members. The coding team conducted initial rounds of thematic analyses which they further developed in thematic memos. They discussed and distilled these themes into codes through a series of team meetings. All team members then applied the code book to the same two transcripts allowing further refinement of the code book and assessment of inter-coder agreement. One or two team members then coded each of the remaining transcripts. The phenomenon of dichotomous labeling and its impact on youth arose de novo from the data.

#### Mothers’ Data

Qualitative analyses were conducted by a team of 4 researchers with two additional senior investigators (AM and MC) reviewing the codebook and offering insights to common themes between youth and maternal data. Similar processes were followed with initial rounds of thematic analysis resulting in thematic memos which the team discussed and distilled into codes at team meetings. Two transcripts were then coded by all team members resulting in further refinement of the code book and assessment of inter-coder agreement. The remaining transcripts were coded by two team members each. Labeling and its impact on youth was identified as a phenomenon parallel to the labeling theme identified in the youth data during early thematic analysis. The code “Labeling” was defined in parallel to the code used in the youth data. The study teams worked together to examine the fidelity of the code across both youth and mother data. Subsequent memos focused on similarities and differences in youth and mothers’ perspectives about labeling.

## Results

The characteristics of the study sample which include youth (n = 39) and mothers (n = 20) are shown in Table 1. Youth in the study were primarily second generation (64%) with eighty percent having lived in the US their entire life. Approximately, 30% were children of an agriculture worker, with 48% having a mother with less than a high school education. All mothers in the sample are first-generation primarily Spanishspeaking; eighty percent have less than a high school education. The average age of mothers’ immigration was 19.9 years (range: 13–29 years). The average number of children per mother was 3 (range: 1–5).

The research team identified dichotomous labels of “good kids” and “bad kids” de novo in the youth interview data (appendix 1). Both youth and mothers identified a variety of people who impose these labels on adolescents in the community, including teachers, school staff members, school administrators, peers, people living outside of Salinas, family members, and other parents. Both youth and mothers identified that labels imposed in school can follow a young person over many years.

Youth and mothers also noted that labels can be based on a variety of sources. In some cases, an adult or peer witnesses a specific behavior which is labeled as “good” or “bad”. In many cases labels are passed on through word of mouth from trusted sources or via rumors from wider social networks. Labels also arise by association. Youth reported being labeled based on their neighborhood as well as the school they attend. One youth described an alternative school as,

A school for … not dumb kids but like kids that are like not going to meet up to their credits so they’re going to have to have like more help… It’s like high school but it’s, yeah, kind of where the bad kids go.

(13-year-old, US-born girl-identified youth)

“Bad kids” or kids with negative labels might have parents who are perceived to be gang affiliated, on probation, or previously incarcerated. One mother reported,

“I didn’t know what they were up to…they wore red pants…and they dyed their hair red…and they wore a lot of red clothes…because they identified with the Norteños from around here. And…they lack manners. So initially, when they were very little, they played together and visited here. But now that they’ve grown…even their mom dyes her hair red…and red clothing. So, to avoid problems, they stay in their house and we stay in ours. And [my daughter] doesn’t hang out with them or anything.” (53-year-old mother of 4, living in the US for 34 years)

Given this, it was important for mothers to know the parents of their child’s friend. When discussing another friend of her child, this same mother noted,

I also know the parents. I’ve been there and have talked to them, and that’s how you get to know people, right? So, I’m certain these are good parents and the girls are good girls.

In most cases situational labels such as these are largely outside of the youth’s control.

### Defining “Good” and “Bad” Kids

#### Good Kids

Youth and mothers reported that “good kids”, or kids who are “on the right path” avoid delinquent behaviors such as truancy, consumption of drugs or alcohol, and physical fights. They also abstain from sex according to youth and mothers. Some mothers described that “good kids” spend most of their time at home with their families. Mothers also noted that “good kids” are obedient and respectful. One mother reported,

“He helps wash dishes. Ever since he was little, I’ve encouraged him to do something: to clean up, to put his things away. “Help me do the laundry.” He helps me with the soap when I’m washing. He’s always very nice, and he’s always been that way ever since he was little.” (41-year-old mother of 3, living in the US for 20 years)

Participants also described “good” as the absence of “bad”. A mother said,

“But the thing is that the kid doesn’t give me any kind of trouble. He’s a good kid, and his only problem is school performance.” (48-year-old mother of 5, living in the US for 20 years)

“Good kids” communicate frequently with their parents and prefer to spend time with family rather than out with friends. Youth were particularly likely to identify “good kids” as those who participate in sports and in a college prep program in high school. When describing his friends, one youth participant said,

“Right now everything’s going good, I don’t see no signs of their future being harmed…Like I don’t see them starting to think drugs are funny or stuff like that.” (13-year-old boy-identified youth, US born)

Mothers and youth shared similar visions of the trajectories of “good kids.” Mothers focused on the fact that kids “on the right path” would have more choice in their employment in the long run, avoiding the manual labor in the fields that many of these mothers experienced. They also envisioned that “good kids” would have financial stability and may have more opportunities for education. Youth were more likely to specifically mention that “kids on the right path” were college bound and more likely to have a “good job” such as one in which they worked indoors. One youth participant said,

“If you’re good you have a lot more like… I don’t know how to say it, you have a lot more opportunities to do things, but when you’re bad, you know, not doing good you really have like less opportunities to do things, you know, less opportunity to go to college, less opportunity to do school activities, and events, and stuff like that.” (13-year-old, girl-identified youth, US born)

#### Bad Kids

Participants described that “Bad kids” or kids on the wrong path are sexually active and engage in delinquent behavior such as skipping classes, drug and alcohol use, and physical fights. Mothers add that “bad kids” spend a lot of time outside of the home. One mother explained,

“A bad friend is… going [out] with them and… my kid doesn’t go out. His friends are the same way.” (48-year-old mother of 5, living in the US for 20 years)

Mothers and youth both noted that youth are labeled as “bad” through affiliation with friends or family who are in gangs or engage in delinquent behavior. One youth explained,

“If you’re not a gang member but you hang out with them, you’re going to look like one, guilty like by association.” (13-year-old girl-identified youth, US born)

A mother recalled,

“What affected my kids most was when one of their uncles said, “I don’t want to see you with my kids, because your brother was killed for being a gang member…. I don’t want to see you with my kids.” That’s what they said to my son who’s an engineer.” (46-year-old mother of 4, living in the US for 25 years)

Mothers ascribed “bad kid” labels to friends they did not approve of. In some cases, this manifests as encouraging youth to socially isolate from. One mother noted,

“Well, as I said, the [friends that] I know haven’t been negative for her. The ones I don’t know were negative. There was one girl I did meet who I didn’t like at all; I really didn’t want my daughter near her. Because they took her out a lot without my permission. And I told her that I didn’t want that girl near her.” (36-year-old mother of 3, living in the US for 18 years)

Both youth and mothers described the tragedy of making one wrong choice, fearing severe outcomes such as ending up homeless, shot, dead, or in jail; they reported difficulty in relinquishing this label and “turning things around.” One mother said,

“Because there are two paths you can choose, and if you choose drugs, you’ll be on the wrong path…you’ll end up in jail…or you’ll get killed…and I don’t want that. You can be better than that.” (37-year-old mother of 4, living in the for 18 years)

Likewise, one youth said,

“Why do you want to be a gangster? You’re just dying for a color, and you shouldn’t – just – just do good in school and then you could be even more rich, and you could travel a lot. And you could have a big house and everything. And the other people, they end up being homeless or dead in the streets or getting shot.” (13-year-old, boy-identified youth, US born)

To protect their children from “being on the wrong track,” mothers adopt a no-margin-for-error philosophy. One mother recalled giving the following advice to her child,

“You have to picture yourself like you’re going up a ladder…and this ladder doesn’t have any railings for you to hang on to. So, if you lose your balance…you’ll fall!” (36-year-old mother of 3, living in the US for 18 years)

Another mother articulated the fear and motivation underlying this idea, stating,

“If we don’t do it that way, then our kids are going to become gang members, get into drugs…Because they’re going to feel like they’re not important to us or like they don’t matter to us… And if we don’t help them, no one else is going to come along with good intentions and tell them, ‘What you’re doing is wrong.’ Quite the opposite: they’re going to be approached by bad people who say, ‘They don’t love you. Let’s go hang out on the street.’” (33-year-old mother of 4, living in the US for 19 years)

### Impacts of Labeling

#### Exclusion from Peers

Youth talked about how peers who were labeled as “bad” could suddenly be ostracized from their friend groups. One youth said,

“I used to talk to them…they were like good girls, like they just started talking to new people and like they got influenced to skip class and all that. I’m like, ‘Bye,’just like keep them out, like I don’t want to do that.” (13-year-old girl-identified youth, US born)

#### Limits to Educational Opportunities

Negative labeling of youth was also found to limit educational opportunities. One youth participant explained the tenacious nature of a “bad kid” label meant that she felt persistently in danger of losing her place in her school

“I was doing bad at [my middle school] so …they kicked me out of the school and they put me over there and it’s like independent study…. I used to be really bad, like getting into fights and stuff, so that all stays like on your school record. So, if anything happens, even if I confronted someone, they’re like, ‘Mm, she’s notorious for doing that, like we got to get her out of here.’” (13-year-old girl-identified youth, US born)

One mother described a vicious spiral of her son being impacted by poverty, labeled a “school failure”, and being denied participation in sports:

“They demand a lot from the kids, such as asking them to focus on school. But being poor, we can’t do that…My kid wanted to join basketball, but he couldn’t because he’s not doing well in school…But I really wish they gave him the opportunity to play basketball…Spending more time in school would make him care more about it, that would motivate him to do better. But instead, they’re just penalizing him for not doing well in school, and that makes everything worse. That lowers his self-esteem and makes him care less about school.” (48-year-old mother of 5, living in the US for 20 years)

#### Changes to Health-Modifying Behaviors

Youth participants explained that their health protective-behaviors were compromised at times to preserve their “good kid” labels. For example, youth participants reported that teens in the community were hesitant to use birth control or buy condoms for this reason. One youth said,

“Probably because they feel like, that they would get judged like if they like go to the store by themselves to get like condoms, then the people would be like, Oh, you’re too young,’ and, or like they’ll feel judged. Or like if a girl goes to the doctor and gets like birth control they’ll be like, ‘Oh, you shouldn’t be doing that,” and stuff.” (13-year-old girl identified youth, US born)

Another participant saw the avoidance of sexual health conversations among his peers as an indicator of “good friend” group. When asked why the sexual conversations do not come up within his friend group he said,

“I don’t know, I guess we’re just all good kids, we just focus on sports.” (13-year-old boy-identified youth, US born)

Youth participants also associated some behaviors detrimental to health with “bad kids.” One youth states,

“And, yeah, and then, I don’t know, I just started running away, kept on selling drugs, doing all that. The experiences that I have with the law is horrible. I’ve been in handcuffs more than twice, more, Jesus, yeah, I was a mess.” (13-year-old girl-identified youth, US born)

### Proximity Impact

All participants displayed less rigid labeling when the subject was themselves or someone in close social proximity such as their friend or child. Mothers were more likely to describe a “good” kid who did a “bad” thing when they were talking about their own child. When talking about her son, one mother mentioned,

“He’s an only child. But it’s gotten really complicated for me. To see all the changes in him, and to see how he’s doing step by step; it seems like he’s going down instead of up.” (32-year-old mother of 1, living in the US for 18 years)

Mothers and youth also described instances of wanting to challenge a label given to a young person in close social proximity to them. Some youth challenged negative labels within the school setting. One youth challenged the negative labels from school employees on her friends:

“Some staff, like, because I’ve been getting in trouble, they say that [my friends are] probably not good people… because people just see the bad of things on them but if they get to know them, like they’re, they’re amazing people.” (14-year-old girl-identified youth, US born)

Youth also challenged negative labels imposed on their city’s reputation through local and regional news sources or word of mouth, making a point to underscore the positive aspects of their community. Regarding Salinas, one youth said,

It’s a nice place to live but sometimes it’s a little dangerous. It’s a nice place to be at because it has many places you can go to. And it’s a really nice place, sometimes in the mornings, you can see the mountains and it’s really beautiful to just look at it… there’s some bad stuff but there’s too some good stuff it has.

(14-year-old girl-identified youth, born outside the US)

Youth discussed the shortcomings of negative labeling and injustices of reducing Salinas to single narrative, often imposed by an outsider viewpoint. One youth noted,

“I’ve never felt like bad being in a neighborhood, like it can be, like I said, [one neighborhood] where everyone says like, ‘Oh, it’s dangerous,’ but I’ve never really felt like I’m in danger or anything….I think it’s just because I grew up here and I feel like everyone outside of Salinas just like makes it like, just adds like more like than what it actually is.” (13-year-old boy-identified, US born)

## Discussion

This study draws from in-depth interviews with early adolescents and mothers in a predominantly Latinx agricultural community in California to demonstrate the pervasiveness of formal and informal “good” and “bad” labels placed on adolescents and variety of ways that these labels impact youth engagement in education and health protective behaviors. Dichotomous labeling was an emergent theme that arose organically from the data, which reflects the lived experiences of our participants in their community. To this end, taking an inductively qualitative research approach, treating our participants as experts in their community and lived experiences, transforms the research relationship as it challenges hierarchical systems of knowledge, and positions participant as a co-producer of knowledge.

### Challenging labels: holding complex personhood

Interestingly, participants in this study voiced nuances in dichotomous labeling. Participants pushed back on negative labeling when it was imposed on those close to them, such as friends, their own children, or their own community. This phenomenon, what we describe as the “proximity impact,” adds to the limited body of research on the deep nuances of marginalization such as good/bad labeling and calls us to examine when we consider the humanity of individuals. It seems that people, places, and events which are perceived at distance, and not seen as a part of a larger “us” are not extended the same compassion as those within our circle. Per Avery Gordan, Adopting a complex personhood framework and upholding an individual’s humanity means to recognize that “people remember and forget, are beset by contradiction, and recognize and misrecognize themselves and others” [18]. Together, challenging dichotomous labeling for those perceived close to us suggest that complex personhood is better practiced for those who are socially connected. Likewise, this phenomenon offers compelling evidence to foster a sense of belonging and strengthen relationships and connections among school personnel, students, families, and community members.

### Labeling as an exclusionary practice and connected to punitive consequences

Participants described the negative labeling of youth as an exclusionary practice that jeopardizes supportive relationships among peers and adults, community connectedness, and school engagement. These findings are in alignment with prior studies on the impact of school disciplinary practices and policies, which disproportionately target students of color [23,24] In such cases, school labels, operationalized through expulsion, suspension, or disciplinary action, follow students’ long term. Studies have shown that being pushed out of school results in a cascade of negative outcomes that ultimately increases the risk for involvement in the criminal justice system [25-27]. Less education has also been highly correlated with poor mental health outcomes and increase health risk behaviors such as smoking, unhealthy eating habits, and being sedentary [28, 29].

Our participants named both the short-term and long-term consequences of the exclusionary labels they witnessed and experienced. In the short-term participants described denial from sports, academic disengagement, school removal, and health-modifying behaviors in the form of drug use and hesitancy in birth control and condom use. Long-term consequences were described as undesired pregnancy, incarceration, gang involvement, and being shot or killed. There is very limited research that examines the public health implications associated with being labeled or avoiding a label, meriting further investigation.

Social labels serve as a cultural and cognitive resource people use to define and categorize the world they navigate. However, social labels prescribed onto individuals must be examined within the living context and social circumstances. Education researcher Shawn Ginwright summarizes this and states, “While it’s true that the conditions of our lives are different, we should be careful not to confuse the quality of conditions of a human with the quality of the human themselves”[30]. Rather than viewing labeling solely through its individual harm, it is necessary to look at labeling through the quality of the conditions, such as the structural environment, that give rise to this phenomenon. More research is needed to better understand the disparate association between punitive and exclusionary labels and low wealth, socio-politically oppressed communities to create source-driven and sustainable solutions.

### Labeling: Responding to oppression

Both youth and mothers in this study emphasized the importance of “staying on the right path” and the literal loss of life and future opportunity for young people who receive stigmatizing labels. One could hypothesize that strict conception of “good” and “bad” is one form of no-margin-for-error messaging given conditions of over-policing and criminalization of youth of color compared to white youth [31].

Oppression is rooted in power imbalances, where a social group or institution with accumulated privileges–such as social capital and material resources–exercises power over another group [32]. In the United States (US), Latinx immigrant families have historically experienced oppression through nativism, racism and ethnocentrism, and are currently living through one of the most anti-immigrant periods in the modern US history with a rise in anti-immigrant rhetoric, laws and policies [33-35]. Oppressive conditions generate negative consequences in several domains: the collective (reduced opportunities, economic disadvantage, and discrimination), relational (isolation, competition across groups), and personal levels (low self-esteem, lack of control) [32]. From a critical pedagogical and liberation psychology lens, oppressive conditions, such as systemic and structural forces that promote sociopolitical injustices, poverty, and resource scarcity, give rise to psychological patterns that often reinforce conditions of internalized oppression [36, 37]. As noted, one mother described a ‘ladder without railings’ articulating a sentiment of there being no room for mistakes, a prevailing message echoed from several youth and mothers in our study. More so, this phenomenon demonstrates community perseverance amidst socially unjust conditions, one way to keep youth from falling off the ladder.

Literature on “legal consciousness,” a perspective infused most fundamentally with stigma and fear, describes internalization of immigration status among first-generation undocumented immigrants. Given the constant reminders of their criminalized presence, undocumented immigrants internalized the most appalling effects of the law that devalue their humanity [38]. Immigration status as a form of labeling powerfully informs how people see themselves and their rights in the United States, internalizing the notion that they have no rights. Over the last decade, as hate crimes against Latinx immigrants gained increasing visibility in the setting of federal immigration raids, anti-immigrant political rhetoric, and xenophobic public policy, legal consciousness is claiming prominence ^[39]^. Like the role of immigration status seen in legal consciousness, fear and stigma predominates in bad/good labeling. Likewise, youth with negative label are at risk of internalizing that label and staying on the “bad” path as described by our participants. To this end, it is possible to address internalized labels harbored by marginalized communities and mobilize a path to liberation by targeting and mitigating causes of stigma and fear and dismantling the conditions that uphold these conditions.

### Public health's capacity to reframe community narratives

The participants in our study challenged negative labels imposed on Salinas, making it point to contrast these labels with positive attributes of their community. This idea arose throughout our study design and implementation with our community-based partners as well. This supports the literature on the insidious impact of deficit-based approaches in public health and research. In “Suspending Damage: A Letter to Communities” Eve Tuck calls for a mortarium on damage-centered research where depletion, brokenness, and hopelessness are used to leverage concern, resources, and reparations for marginalized communities [42]. Designation terms used in research and public health to label oppressed communities and youth such as “resource-scarce”, “poverty-stricken” and “at-risk” pathologizes and romanticizes a single narrative of suffering that curbs trust and relationship-building with these communities [41, 42]. Instead, Tuck calls for researchers and public health practitioners to capture a desired rather than a damaged-based framework that incorporates complex personhood and is “concerned with understanding complexity contradiction, and self-determination of lived lives” [43]. Our participants echo this call to suspend unidimensional damaged-centered narratives imposed on their communities.

A potential strategy to combat this deficit-centered labeling of communities like Salinas is asset-framing – which emphasizes the strengths, aspirations and dreams over challenges and the benefits of investing in these communities for positive change [44]. More research rooted in asset-based framing is needed not only to better to assess its potential as an advocacy tool, but also equitably target structural and systemic causes of oppression while still capturing complex personhood and humanity of communities of color.

Another avenue to reframe the deficits-focused narrative is rethinking the way research is conducted and analyzed. Our analysis offers an example of a more nuanced insight into labeling beyond mapping the harmful consequences of “good” and “bad”, where this dichotomous labeling can be viewed as an act of community perseverance under oppressive conditions. Likewise, remedying the harmful consequences of interpersonal and community labeling requires deconstructing the power that give rise to oppressive conditions through everyday societal structures and institutions.

Rich (2017), primarily focusing on homelessness, posits that removing harmful labels requires us to focus on remedying structural challenges such as limited resources, increased police presence in schools with predominantly minoritized youth, community violence, understaffing in school, and overcrowding [45]. Along these lines, examples of structural and institutional change are getting rid of zero-tolerance school policies, reimagining true authentic safety as community-engaged and trauma-informed, promoting healing-centered practices as opposed to policing and surveillance, and considering alternative practices to addressing disruptive behavior that relies on conflict resolution, peer mediation and restorative justice [46-49].

## Conclusions

This study extends Labeling Theory to understand the public health implications of labeling adolescents within a primarily Latinx agricultural community. Formal and informal “good” and “bad” labeling of youth had implications for youth engagement and health-protective behaviors and educational pursuits. Social connection allowed youth and parents to challenge negative labels, which offers compelling evidence against exclusionary practices and in favor of practices that foster a sense of belonging and strengthen connections among school personnel, students, families, and community members. Our participants also highlighted the harm in damaged-centered community narratives prescribed to communities like Salinas, calling us to consider how to incorporate more asset-framing and desired-center frameworks in public health work. This study’s findings can help public health practitioners better understand the perceptions and experiences of youth from immigrant and Latinx communities, which can aid in optimizing public health interventions that seek to improve the health, well-being, and academic success of youth.

## Figures and Tables

**Table 1:** Characteristics of youth and mother participants

	Children (N = 39)		Mothers (N = 20)		
	N	%	N	%	Mean (range)
*Self-identified gender*					
Boy	19	48.7			
Girl	20	51.3	20	100	
*immigrant generation*					
1st gen	8	20.5	20	100	
2nd gen	25	64.1			
3rd gen +	5	12.8			
Unknown	1	2.6			
*Years lived in the US*					
Entire life	30	76.9			
More than 5	7	17.9	20	100	21.1 (14,34)
5 or fewer	2	5.1			
*Age at Immigration*					
Less than 18 years old			5	25	
18 years or older			15	75	
Average age (range)					19.9 (13,29)
*Mother’s education*					
Less than high school	19	48.7	16	80	
High school only	11	28.2	1	5	
Post-High school	9	23.1	3	15	
*At least one parent in Ag*					
Mother works in ag	15	38.5	15	75	
Working, other			3	15	
Not working			2	10	
*Living situation*					
Both parents	22	56.4			
Mother only	14	35.9			
Other	3	7.7			
Number of children					3.4 (1,5)

## Data Availability

The datasets generated and/or analyzed during the current study are not publicly available to protect the privacy of study participants but are available from the corresponding author upon reasonable request
